# Statistical Amplification of the Effects of Weak Magnetic Fields in Cellular Translation

**DOI:** 10.3390/cells12050724

**Published:** 2023-02-24

**Authors:** Vladimir N. Binhi

**Affiliations:** Prokhorov General Physics Institute of the Russian Academy of Sciences, 38 Vavilov St., 119991 Moscow, Russia; vnbin@mail.ru

**Keywords:** biological effect of magnetic field, ribosome, protein translation, incorporation error, the RPM, geomagnetic field

## Abstract

We assume that the enzymatic processes of recognition of amino acids and their addition to the synthesized molecule in cellular translation include the formation of intermediate pairs of radicals with spin-correlated electrons. The mathematical model presented describes the changes in the probability of incorrectly synthesized molecules in response to a change in the external weak magnetic field. A relatively high chance of errors has been shown to arise from the statistical enhancement of the low probability of local incorporation errors. This statistical mechanism does not require a long thermal relaxation time of electron spins of about 1 μs—a conjecture often used to match theoretical models of magnetoreception with experiments. The statistical mechanism allows for experimental verification by testing the usual Radical Pair Mechanism properties. In addition, this mechanism localizes the site where magnetic effects originate, the ribosome, which makes it possible to verify it by biochemical methods. This mechanism predicts a random nature of the nonspecific effects caused by weak and hypomagnetic fields and agrees with the diversity of biological responses to a weak magnetic field.

## 1. Introduction

An extensive literature [1,2,3,4] is devoted to the biological effects of weak magnetic fields (MFs). As is believed, MF can cause various, including toxic, effects in organisms [5]. The difficulty lies in the fact that there is no convincing physical mechanism that would provide a discernible shift in the probability of an individual act of a chemical reaction in the MF on the order of the geomagnetic field, about 50μT [6]. One of the mechanisms relates the biological effect of MFs to the presence of magnetic nanoparticles in organisms [7,8]. However, some cell cultures and plants that respond to MFs do not contain magnetic nanoparticles. Therefore, search for a general molecular mechanism of the biological response to MFs continues.

The characteristic of the current state of the problem is that even the most plausible mechanism of magnetic biological effects, the Radical Pair Mechanism (RPM), provides only an insignificant, at best on the order of 0.01–0.1%, response to a change in the MF of the geomagnetic field level [9,10]. Several orders of magnitude are missing to explain the facts of animal magnetic navigation [6]. The difficulty is that the coherence of spin quantum states, which provides the magnetic effect at physiological temperatures, takes place over a short time interval. In weak MFs, however, a noticeable effect occurs only at a sufficiently long coherence relaxation time. These conflicting trends prevent the occurrence of a magnetic RPM effect sufficient to explain the observations. Noticeable magnetic effects in magnetochemistry usually arise in MFs exceeding 5 mT [11]. Accordingly, as is shown below, the thermal relaxation time of electron spins in biological media at physiological temperature should be about 1 ns in order of magnitude. At the same time, to explain the biological effects of MFs of the geomagnetic field level of 0.05 mT, the thermal relaxation time of electron spins must exceed a few hundred ns, i.e., be comparable with the Larmor precession period of about 700 ns. It is unknown where and whether such spin states could appear in biological tissue.

Apparently, the small primary changes that occur as a result of the action of MF must somehow be amplified to cause noticeable changes in concentrations of biochemical agents. This idea is not new. The enzymatic reaction, highly responsive to the enzyme concentration, was proposed in [12]. A chemical reaction in a mode close to bifurcation instability, when the reaction path can change dramatically with a slight variation in the reactants content, was proposed in [13,14,15]. However, these approaches have not been developed—probably due to the impossibility of their experimental verification. The idea was put forward of a statistical amplification of parallel negligible magnetic signals from millions of photoreceptors by the brain. However, the gain appeared to be insufficient to explain the magnetic navigation of animals based on the RPM [16]. Moreover, this mechanism does not apply to nonspecific effects—in the absence of evolutionarily developed magnetoreceptors.

In this paper, we pay attention to the fact that small initial signals can be statistically amplified—in the course of their accumulation in a long series of repeated events. It is known, for example, that a small physical effect of the effort of thought can become noticeable after millions of elementary acts of scattering in the quincunx or acts of generation of binary random events. The integrated deviation served as a tool for detecting the effect—as a tool that allowed one to accumulate minor regular deviations against the background of significant random variations, see, e.g., [17] p. 319. In the same way, one would expect that magnetic effects, if they are due to primary minor signals under the action of an MF, could be reliably detected where they accumulate in a long sequence of elementary events.

The natural carrier of such an integrator in the cell is the process of gene expression. The essence of these processes is the multiple and almost error-free repetition of homogeneous acts of biochemical reactions involving biopolymers. Such cyclic processes are ideal integrators; they accumulate the probabilities of errors occurring in each step. Sooner or later, the accumulation of error probabilities leads to disruption of the functions of the synthesized and folding proteins, which one can record in the experiment. Therefore, a relatively stable magnetic effect is mainly expected where there are intensive processes of replication, transcription, translation, and folding—in cells under radiation or chemical stress.

There are almost no data on the sensitivity of pre-biological systems to a weak MF in vitro. Additionally, if there are [18], then their relationship with the effects in vivo remains questionable [19]. If we do not consider the evolutionarily fixed magnetic sensitivity in seasonally migratory species, then marked and reproducible MF effects in biology occur in systems with intense gene expression. Such experimental observations include morphological changes during embryogenesis [20], neurite outgrowth [21], restoration of a severed head in planarians [22], response to heat shock [23], some phases of cell growth and gene expression in plants [24,25,26], effects of ionizing radiation [27], a direct controle of the DNA synthesis with magnetic ions [28], etc.

It follows gene expression is almost mandatory for the observation of magnetic responses. Numerous data on the strong dependence of magnetic effects in cells on their genetic modifications also make it evident. All those works indicate that gene expression, which includes various processes of biopolymer synthesis, may be a prerequisite for the MF nonspecific effects.

It is essential that cyclic processes of biopolymer synthesis are catalytic. They occur due to enzymes that directly produce elongation of the biopolymer chain. On the one hand, in such cyclic processes, the low probability of an error in one cycle accumulates and leads to a significant chance of functionality violations of the synthesized biopolymer. Other enzymes that do not elongate biopolymers do not have this feature—the low probability of error in a single act means an equally small fraction of erroneous product molecules. On the other hand, intermediate states of paired radicals with spin-correlated electrons can arise in the active sites of enzymes, e.g., [29,30]. Such quantum states are magnetically sensitive.

The effect of MFs on enzymatic activity has been discussed for a long time. For example, in [31], 2-T MF caused a change in carboxydismutase activity from approximately 9 to 30%. The change occurred when the enzyme-substrate mixture was exposed to MF from 1 to 192 h. In [32], MF of 5 T did not cause statistically significant changes in DNA hydrolysis by ribonuclease and reduction in cytochrome-c by succinate-cytochrome-c reductase. No noticeable changes in the activity of ribonuclease, polyphenol oxidase, horseradish peroxidase, and aldolase were observed in [33] upon exposure of the enzyme-substrate mixture for up to 20 min to an MF of up to 17 T. It was shown in [34] that a 30 min exposure to an MF of 1.1 T caused a 12% decrease in the activity of ascorbic acid oxidase. In [35], MF affected the synthesis of adenosine triphosphate by creatine kinase with ${}^{25}$Mg${}^{2+}$ ions in the catalytic centers. The rate of synthesis increased by 70% in the field of 80 mT. Replicative experiments are also known, which failed to confirm the effect of MF on the activity of creatine kinase [36].

More recent works have not made effects of MFs on enzyme activity clearer. In [37], an MF with a value slightly less than 1 mT could enhance or suppress the activities of superoxide dismutase and catalase in radish seedlings, depending on the lighting conditions. The effect of an MF of 10–160 mT for up to 20 s on several redox flavoprotein enzymes was studied in [38]; no changes were found. An MF of up to 500 mT and a duration of up to 3 h changed lipase activity for up to 50% in [39]; there was an increase or a decrease depending on the MF and other conditions. In [40], a 10% activity growth of fungal laccase was observed in a variable magnetic field of about 17 mT in the frequency range of 10–50 Hz. The effect of a static MF of up to 220 mT on the enzymatic DNA synthesis in the presence of magnesium ions was demonstrated in [41]; the activity of DNA polymerase in this study decreased by 2–4 times. The review [26] cites many other observations of MF-modulated enzyme activity.

In general, these studies do not show any common pattern that controls the occurrence of the magnetic effect or links its magnitude with the parameters of magnetic exposure, especially in in vivo studies. The MF effect on enzymatic activity appears to be largely random. Apparently, the changes that occur at the molecular level—when they occur—depend significantly and ambiguously on many biochemical conditions and affect, also ambiguously, the measured characteristics [42].

Thus, on the one hand, the premises taken into account in this work are as follows. It is most likely that (i) the biological effects of a weak MF originate from the MF action on gene expression, and (ii) the primary mechanism for the weak MF effects in organisms is the RPM. On the other hand, the facts that one should interpret are (a) the observed nonspecific biological effects are orders of magnitude greater than the primary RPM signals that occur in response to a weak MF, and hence some amplification must exist of the primary signals; and (b) in the vast majority of cases, these observed effects are random.

The purpose of this work was to build a statistical model of the accumulation of small primary signals in the probability of local translation errors under the action of a weak MF up to a level of more than a few percent. The process of local error amplification and the RPM are combined into a single model to evaluate a much greater possible magnetic effect. Such a mechanism would then explain the observed effects of weak MFs without contradictions.

In the next section, we present the biochemical information that underpins the model presented in section Mathematical Model. In the Discussion, we analyze the properties of the model and discuss the consequences arising from it. We show that this statistical model has the properties necessary to explain the specific magnetoreception and nonspecific magnetic response. Section Appendix A describes the RPM in a simple form, which is embedded in the model.

## 2. Amplification of the Local Error Probability in Cellular Translation

The processes of biopolymer synthesis are diverse. These are DNA replication, transcription—synthesis of complementary RNA, splicing, translation—protein synthesis from amino acids under the mRNA code, and post-translational folding of the protein chain into a globule and its maturation. At each stage, random errors can occur, but with significantly different probabilities. There are perfect biochemical mechanisms for correcting replication and transcription errors; the likelihood of these errors is therefore tiny, on the order of $10^{-8}$ and $10^{-5}$, respectively, [43]. Translation errors occur much more frequently, with a probability of the order of $q=10^{-4}$–$10^{-3}$ per added amino acid [44,45]. Then about $1-{\left(1-q\right)}^{300}∼3$–26% of synthesized molecules of 300 units contain at least one error. For such molecules, the chance of adopting a native conformation during folding significantly reduces. Often they are cytotoxic and cause harmful cellular effects [46].

Errors also occur at the folding stage—one of the causes is the intricate geometry and topological nodes of the folding trajectories [47]. However, misfolding due to translation errors, as is believed, is more likely than due to the actual folding. Therefore, translation errors mainly control the accuracy of gene expression. Consequently, the possible influence of MFs on the probability of translation errors is the process where magnetic effects could manifest themselves at the biological level. However, the possibility of MF influence on translation errors, as far as we know, was not previously considered in theoretical models.

The translation is a complex multi-stage cyclic process that includes a variety of enzymatic reactions. The ribosome produces translation—it is a macromolecular machine assembling amino acids into proteins. Below is a statistical model of ribosomal translation in which there is a low probability of a local incorporation, or substitution, error—the appearance, in the synthesized chain, of a non-cognate amino acid residue that does not correspond to the mRNA blueprint.

A simplification illustrating the occurrence of a noticeable translation error is as follows. Let a ribosome synthesize a protein chain of a large number *n* of links with an equal probability ξ of incorporation error. A native functional protein globule implies the absence of local errors at all *n* links in the chain. Then ${\left(1-\xi \right)}^{n}$ is the probability of occurrence of error-free amino acid sequence, and

(1) $$p=1-{\left(1-\xi \right)}^{n}$$

is the probability of the appearance of a defective molecule, i.e., the probability of a translation error. The sensitivity of *p* to the local incorporation error probability ξ is the derivative $dp/d\xi =n{\left(1-\xi \right)}^{n-1}$. Its magnitude is maximum under the condition $n\approx {\left(2\xi \right)}^{-1}$ and can reach large values of about ${\left(4\xi \right)}^{-1}$ at small ξ.

The probability of correct translation of the entire molecule does not exceed unity. In particular, for nξ∼1 and n≫1 we have p∼1−1/e≈0.63. This fact means that the result of changing the error probability when ξ varies is not that the probability (1) changes much, but that a change in the translation error *p* by a few tenths occurs when varying very small ξ.

The number of different proteins in the human body exceeds two million [48]. Most of them have a length of 100 to 500 amino acids. The probability of a local failure ξ is unlikely to reach $10^{-2}$ because almost all proteins would fold incorrectly otherwise. Therefore, the range $\xi <10^{-3}$ is interesting.

Since small probabilities ξ of substitution errors lead to significant variations in the translation error, it makes sense to assume that MF can change those probabilities of local errors. Then, a magnetic effect, even being minor initially, could manifest itself in significant changes in the concentration of nonfunctional proteins. These, in turn, would lead to an additional load on biochemical adaptation mechanisms and the appearance of noticeable biological effects.

How can MF affect the probability of local incorporation errors ξ? Cyclic processes of biopolymer synthesis are those produced by enzymes that elongate the biopolymer chain. In translation, the ribosome cyclically reads information from mRNA and attaches a suitable amino acid to the synthesized protein chain. We suggest that this enzymatic recognition/attachment in the ribosomal active site includes the electron transfer and formation of the intermediate radical pair in the singlet spin state. If a necessary amino acid enters the active site, then the process ends with chain elongation without error. If a non-cognate amino acid gets into the active site, this amino acid is not accepted. The radical pair decomposes into the initial state of the enzyme and amino acid, and the latter goes out into the cytoplasm.

The picture in more detail is as follows. Protein biosynthesis is known to consist of two steps, e.g., [49]. First is the extraribosomal stage. In it, the enzymatic selection of amino acids occurs by attaching them to the corresponding specific transfer RNA—aminoacylation of tRNA by the enzyme aminoacyl-tRNA synthetase. Then the tRNA with a cognate amino acid enters the ribosome, where a comparison occurs of the specific tRNA with the instruction of the messenger mRNA. If there is a match, the enzyme detaches amino acid from the tRNA and attaches it to the end of the synthesized chain. If not, the tRNA-amino acid complex disintegrates and escapes into the cytoplasm. All these processes, in turn, can go in several stages. It is not yet clear at what stage the emergence of an intermediate magnetosensitive radical pair is more likely. Therefore, we consider the following general model regardless of where the radicals appear. The proposed kinetic scheme, Appendix A.1, combines the processes of different stages and distinguishes among them one that hypothetically occurs with the formation of an intermediate state—a temporary pair of radicals—and can end with an error.

When developing the RPM-based kinetics, the MF is usually assumed to affect the radical pairs that arise in a “correct” biochemical reaction that leads to the formation of valid product molecules. Indeed, in the case of reagents that are not biopolymers, the likelihood of a product that is chemically different from the expected one is negligible, if at all possible. Another situation occurs in cyclic reactions involving biopolymers. Here, the occurrence of errors is the norm [50] and even necessary to ensure biological evolution [46]. In this case, it is reasonable to assume that the MF can influence such a process through the RPM.

As mentioned above, the RPM is now considered the most likely molecular basis for magnetic effects in biology [3,51]. The fact that radical pairs often arise in enzymatic reactions supports this view. According to the RPM, an external MF causes a singlet-triplet (S-T) conversion in the spin state of the initial singlet radical pair and, thereby, reduces the probability of its decomposition. Then, there occurs a possibility of including an incorrect amino acid group in the protein chain. A local incorporation error appears. Thus, MF increases the likelihood of the appearance of defective protein chains that cannot further acquire the correct conformation or become functional globules.

## 3. Mathematical Model

In a realistic scenario, independent random variables $\xi_{i}$, i=1,2,...,n on each link represent the failure probabilities. Let all of them have the same distribution with expectation $E[\xi_{i}]=\zeta$ and variance $D\left[\xi_{i}\right]=\sigma^{2}$. In this case, the translation error

$$p=1-\prod_{i=1}^{n}\left(1-\xi_{i}\right)$$

becomes a random variable with mean P=E[p] and variance $S^{2}=D\left[p\right]$, respectively

(2) $$P=1-{\left(1-\zeta \right)}^{n},\;\;S^{2}={\left[\sigma^{2}+{\left(1-\zeta \right)}^{2}\right]}^{n}-{\left(1-\zeta \right)}^{2n}$$

Expanding (2) into a series in ζn, one can see that in the region of large *n* and small ζ<1/n the relations P≈ζn and $S\approx \sigma \sqrt{n}$ take place. The average incorporation error ζ increases by a factor of *n*, and the fractional value S/P of variations decreases as $1/\sqrt{n}$. Since the length of most proteins is about a hundred and more, the main amplification effects arise from the change in the average value of the errors and not from their variations. Next, we estimate ζ and its dependence on the MF.

To analyze the dependence of average probability *P* of a translation error on MF *H*, it is necessary to sequentially relate MF *H* to the S-T conversion rate ω(H), then relate rate ω to the incorporation error rate u(ω), further relate rate *u* to the probability ζ of an incorporation error, and finally relate probability ζ to the translation error probability P(ζ). In what follows, it will be convenient to use dimensionless quantity $h\equiv H/H_{g}$, i.e., MF *H* in units of the geomagnetic field $H_{g}\approx 0.45$ Oe that corresponds to the magnetic flux density of 45 μT. All necessary relations look like h→ω→u→ζ→P.

The regularities of S-T conversion in the spin state of a pair of radical electrons give the first dependence ω(h). The equation—in a simplified form—relating the rate of the S-T conversion with an external MF, is

(3) $$\omega \left(h,\tau \right)\propto \frac{1}{\tau }\left[a+z\left(\tau \right)h^{2}\right]$$

where *a* is a constant part, $z\left(\tau \right)\equiv \gamma^{2}H_{g}^{2}\tau^{2}$ is a model parameter, $\gamma =1.761\times 10^{7}$ G${}^{-1}$ s${}^{-1}$ is the gyromagnetic ratio of the electron, and τ is the time of its spin decoherence, $z\left(1\;\mathrm{ns}\right)=7.752\times 10^{-5}$, Appendix A.2. The rate of S-T conversion is a quadratic function of *h*, which means that the magnetic effect does not change at the MF reversal. The spin decoherence time is yet unknown at physiological temperature and a zero-field condition. This condition is relevant because the MFs in this study are less than the hyperfine MFs by orders of magnitude. For this reason, we consider τ a model parameter that can vary in a wide range, from usual spin-chemical values of about 1 ns to hypothetical values of about 1 μs.

The second dependence u(ω) is derived from the equations of chemical kinetics, and has the form of Equation (A3), see Appendix A.1. With regard to the third dependence, we note that probability ζ of an incorporation error upon elongation by one peptide bond is determined by the kinetic rate of occurrence of errors *u*. Since elongation by one peptide bond occurs in the time Δt, then ζ=uΔt.

Finally, Equation (2) determines dependence P(ζ), whence, substituting the above relations for *u*, ζ, and ω and taking into account that $u_{0}=q/\Delta t$, where $q=10^{-4}$–$10^{-3}$ is the experimental probability of incorporation error per peptide bond, we write

(4) $$P\left(h\right)=1-{\left[1-q\;\frac{1+j\tau /[a+z(\tau )]}{1+j\tau /[a+z\left(\tau \right)\;h^{2}]}\right]}^{n}$$

In view of Equation (3), it is convenient to use dimensionless parameters J≡jτ/z(τ) and A≡a/z(τ). With these parameters, the translation error probability has the form

(5) $$P^{\prime }\left(h\right)=1-{\left[1-q\;\frac{1+J/(A+1)}{1+J/(A+h^{2})}\right]}^{n}$$

independent of the spin relaxation time.

**Figure 1:**
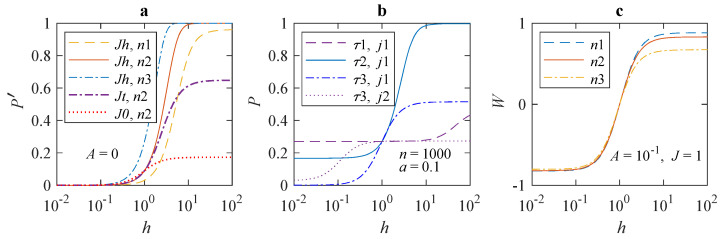
The dependences of translation error probability on the MF $h\equiv H/H_{g}$ for different values of the model parameters: (**a**) dependence (5) at different length *n* of the synthesized protein molecule, n1=100, n2=300, and n3=1000 and different relative chemical parameter *J*, J0=1, Jt=10, and Jh=100; (**b**) dependence (4) for different values of τ, $\tau 1=10^{-9}$ s, $\tau 2=3\times 10^{-8}$ s, $\tau 3=10^{-6}$ s and different values of the chemical parameter *j*, $j1=10^{8}$ 1/s, $j2=10^{6}$ 1/s; (**c**) relative magnetic effect W(h)≡[P(h)−P(1)]/P(1) at different length *n* of the synthesized molecule. MF $h=10^{0}=1$ corresponds to the geomagnetic field.

Figure 1 shows dependences of the average error probability in different forms on MF $h\equiv H/H_{g}$ in the *H* range from 450 nT to 4.5 mT. The features of the dependences are clearly visible on the logarithmic scale of the MF. It can be seen from Figure 1a that the change in the error probability $P^{\prime }$ with increasing MF reaches significant values, far exceeding what is usually observed in magnetochemistry in such weak MFs. The magnitude of deviations increases with the length *n* of a synthesized chain. The position of the inflection region depends little on *n*, shifting towards smaller fields with increasing n.

Figure 1b shows the shift of the inflection region in *P* for absolute values of parameters *a* and *j* as τ changes. The inflection region shifts approximately from 100 *h* to 1 *h* towards lower MFs with an increase in τ by three orders. It shifts even more at a lesser *j*. In other words, there is a wide range of τ values, in which the inflection region shifts directly with τ. Therefore, one could determine an unknown value of τ from dependence P(h) obtained experimentally.

In the experiment, one usually works with normalized relative effects. The magnitude of a measured value in the geomagnetic field, i.e., at h=1, often serves as “control”. Since the level P(1) of error in the geomagnetic field also depends on the parameters, the dependence of normalized effects on the MF may differ from P(h). It is natural to define normalized magnetic effect as the fractional difference between the error probabilities in the MF *h* and in $h_{g}=1$, i.e., W(h)≡[P(h)−P(1)]/P(1), Figure 1c. It can be seen that the relative effect is larger, depends little on *n*, and has opposite signs, ±, in fields greater than and less than the geomagnetic one. The magnetic effect saturates both with increasing and decreasing MF.

In the region of large MF values, the relative effect is weaker for longer chains, which might seem counterintuitive. This is because both P(h) and P(1) depend on *n*. To explain the decrease in *W* is easier by considering the relative effect in terms of expansion P(ξ)≈ξn shown above. A more exact expansion of *P* in (2) reads $P\left(\xi \right)\approx \xi n-\xi^{2}n^{2}/2$. As *n* grows, the negative quadratic term in *P* starts to play a role, and this term is larger, as seen, for larger ξ. Since ξ monotonously increases with *h*, the relative effect *W* weakens with *n*. For small *n*, W(h) for any *h* reaches a limit that has no practical significance, since the absolute magnetic effect *P*, Figure 1b, tends to a value of the order of *q*, which means that there is no amplification.

**Figure 2:**
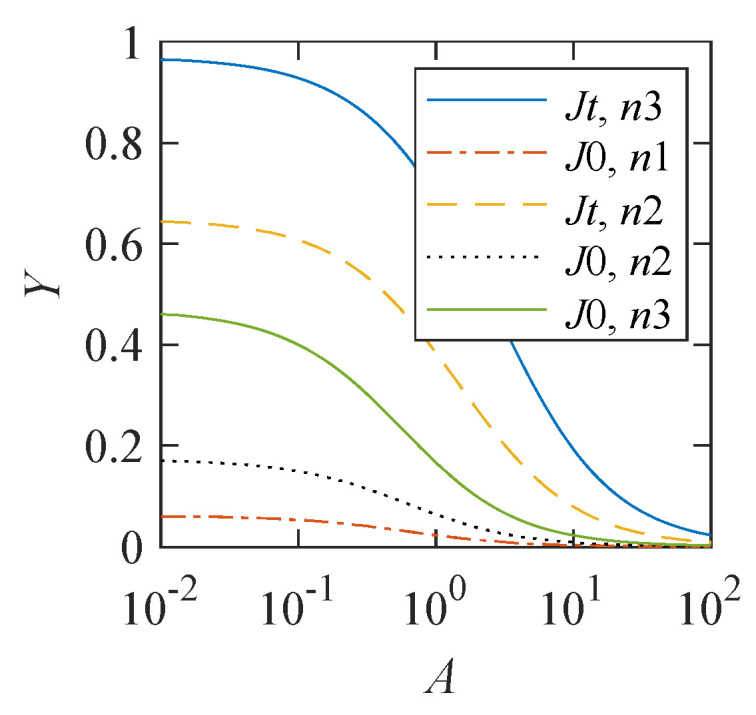
Dependence of the span $Y\left(A\right)\equiv P^{\prime }\left(\infty \right)-P^{\prime }\left(0\right)$ of the magnetic effect on *A*—a relative value of parameter *a* in (3)—at different values of the chemical constant *J* and different lengths *n* of the synthesized protein chain.

As follows from (3), the translation error probability depends on the ratio of the rate parameters *a* and z(τ), or A≡a/z(τ). To represent this dependence, the span of the magnetic effect, defined as $Y\left(A\right)\equiv P^{\prime }\left(\infty \right)-P^{\prime }\left(0\right)$, is used. Figure 2 demonstrates that this dependence is significant. The magnitude of the magnetic effect decreases with an increase in the relative value *A* of the constant component in the S-T conversion rate. However, when the model parameters n>300 and J>10, a 10% magnetic effect appears even at a relatively large value of A=10.

## 4. Discussion

First, we emphasize that formula (4) describes the accumulation of probabilities, not physical changes. Physical changes happen suddenly at a random moment in translation. The transformation of small probabilities into a significant one does not occur in time as the chain elongates. It refers to the result of the synthesis—an entire molecule.

Figure 2 demonstrates that the constant *a*, or *A* in dimensionless form, is crucial for the observability of magnetic effects. For A≪1, there is a convincing dependence of the translation error probability on the MF, while for A≫1, there is practically no such dependence. Equation (A5), $w\left(h\right)\propto a+z\left(\tau \right)\;h^{2}$, from which the S-T conversion rate (3) follows, establishes an observability criterion for magnetic effects

(6) $$a\ll z\left(\tau \right)\;h^{2}$$

in order of magnitude in the region h∼1. If a≫z(τ), the relative magnetic effect in the MFs of the geomagnetic field level will be much less than unity. Additionally, this means the practical impossibility of observing the magnetic effect in biochemical or biological quantities due to the usually high random fluctuations in these quantities. The amplifying mechanism only partially removes this limitation since, while strengthening the absolute values of the probabilities, it can only weaken the relative values when absolute ones approach unity.

For example, in the RPM, the probability of a triplet product in small MFs, much smaller than the HFI field, has, according to [52], the form of Equation (A4). For small values of the master parameter ${\left(\gamma H\tau \right)}^{2}$, the probability of a triplet product is proportional to $1+{\left(\gamma H\tau \right)}^{2}/6$, i.e., a∼1. Value A≡a/z(τ), in this case, can be very large, depending on the relaxation time τ.

There are only two ways to overcome the strict constraint imposed by the observability criterion (6). One should assume that either the spin coherence time τ is large or the constant *a* is sufficiently small. The RPM models of animal magnetic navigation use the first assumption as a rule. Even in this case, a reliable explanation fails since, in the case of magnetic navigation, the characteristic value of the MF is not the geomagnetic field but a thousand times lower geomagnetic variations ΔH of the order of tens of nT. It is these variations that some animals detect and use to survive in seasonal migrations. This being so, $w\left(H_{g}+\Delta H\right)\propto a+{\left(\gamma H_{g}\tau \right)}^{2}+\Delta w$, where Δw=ΔH(dw/dH). Even for τ∼1 μs, the increment Δw remains small compared to the constant component, which is now $a+{\left(\gamma H_{g}\tau \right)}^{2}$. The ratio of these two is about $10^{-3}$ for a∼1. That is, the requirement of the smallness of the constant component in *w* is far from being fulfilled.

It is yet unclear whether small values of *a* satisfying (6) are possible. Small *a* would mean that some microscopic physical quantity in a zero external MF has only a fluctuation component. S-T transitions in the RPM do not satisfy this condition since they also happen in the absence of external MF—under the influence of sufficiently strong fields of the nearest magnetic nuclei. However, the idea of a play of quantum levels looks attractive. When the levels mix in a zero MF—when quantum selection rules lose their significance—some physical quantities could become very small.

Interestingly, in addition to the RPM, there is another mechanism of the primary magnetic response—the so-called level mixing mechanism which utilizes a dynamics of a single magnetic moment [53,54], rather than that of a pair of moments as in the RMP. This mechanism is very abstract, and it is not yet clear whether it could operate within the enzymatic machine of cellular translation. The probability of an abstract chemical reaction in this mechanism is given by

$$w\left(H,\eta ,\tau \right)=1-\frac{1}{2\pi }\int_{0}^{2\pi }\exp\left\{-\eta \tau \left[1+\mathrm{sinc}\left(\frac{\gamma H\tau }{2}\right)\sin\left(\phi \right)\right]\right\}d\phi$$

where η is the average rate of the biophysical events initiated by the precessing magnetic moment. Can condition (6) be satisfied here? To answer, we need to verify the validity of inequality $w\left(0,\eta ,\tau \right)/w\left(H_{g},\eta ,\tau \right)\ll 1$ that follows from $a\ll z\left(\tau \right)\;h^{2}$. One can show that this ratio does not fall below 0.8 for any parameter values, i.e., condition (6) is not satisfied. This mechanism has the same difficulty as in the RPM—the reaction takes place in zero MF, and changing the MF around the geomagnetic field can alter its rate insignificantly.

Until now, to explain biological magnetic effects on the basis of the RPM, one had to assume a long thermal relaxation time of electron spins in biological tissues, on the order of 100 ns and more [55]. The presented model of a statistical amplification of weak primary magnetic signals is free from this presumption. To calculate the MF dependences Figure 1b, demonstrating an effect sized enough to be observed, thermal relaxation time of electron spins was set equal to 1–10 ns also.

The statistical mechanism of increasing the probability of incorporation errors largely compensates for the lack of the effect size in the RPM. Combining these two mechanisms into a single one makes it possible to explain the relatively large observed magnetic effects at short spin relaxation times, even if the constant part in the S-T conversion rate is relatively high, as seen in Figure 2.

As is known, strong MFs, of the order of 1 T and more, used in magnetic tomography, are safe at limited times of MF action of the order of 20–30 min [56]. This no harmful effects, of course, does not mean the absence of any biological effects of such an MF in general. The above-presented regularities explain why strong MFs exceeding the geomagnetic field by four or more orders of magnitude do not lead to likewise strong magnetic effects in comparison with those in the MF of the geomagnetic level. Due to the kinetic limitations of the RPM, the magnetic effect is saturated already at the molecular level. In the idealized model, saturation occurs in fields approximately an order of magnitude different from the geomagnetic field, reaching about two-fold effects in $h_{g}$. In reality, the values of magnetic alterations measured in biological variables are unlikely to be very large due to various adaptive feedbacks. Due to the presence of systems in cells that destroy misfolded proteins, the final observed effects are unlikely to be as great as the model predicts. An idealized model can explain qualitative rather than quantitative patterns.

In general, it is difficult to predict how the deviation in the level of translation errors from its natural level at $H_{g}$ will affect the observed biological characteristics. It is unclear what is the subsequent transduction pathway of the mistranslation signal to a measured reaction. Even the sign (±) of the body’s response to MF is not clear. For example, an increase in the concentration of reactive oxygen species, in response to an MF change, as a secondary biochemical effect could occur with both an increase and a decrease in MF relative to the natural level to which the body is adapted. Therefore, when discussing the connection between the aberrant translation and the values measured in the experiment, it would be reasonable not to pay attention to the sign of effects but to interpret only the qualitative features of the MF response.

The list of qualitative features includes (a) independence of the direction of the external MF, (b) significant absolute and relative magnitudes of translation errors depending on the weak MF, (c) saturation of the relative magnetic effect both with increasing and decreasing MF from the geomagnetic level, (d) unpredictable sign of the effect observed by secondary changes in the body, i.e., the random nature of the measured effect. (e) Under in vitro conditions, the concentration of the defective product does not depend on the MF frequency in the low-frequency range. In particular, the responses to a constant MF *H* and to a variable one with an amplitude $H\sqrt{2}$ should be the same. Model validation would be possible not only in vivo, but also in vitro—in laboratory biochemical translation systems. They are being actively developed [57]. The dependence of the reaction yield on the MF proves the existence of an intermediate S-T state of a pair of electrons in magnetochemistry. Similarly, observation of the MF-dependent concentration of incorrectly synthesized molecules in vitro would be a direct evidence of the existence of an intermediate S-T state of electron pairs during translation.

In calculations [55], the relative RPM magnetic effect in a simple configuration “two electrons, one proton” was about 10% when the MF changed by the value of the geomagnetic field. At first glance, this is enough to explain the nonspecific effects of the geomagnetic field. The problem, however, is that a long thermal phase relaxation time of electron spins, 1 μs, was used in the calculations. The authors of [58,59] attempted to substantiate such a great value theoretically. However, there are no experiments so far that would confirm the existence of this long relaxation time, with the exception of exotic systems, such as fullerenes, which have nothing to do with biology. Reliable evidence of the spin relaxation time in radical pairs could be given by measurements of the EPR linewidth in the geomagnetic field or by measurements of the spin magnetic effects in vitro and their comparison with calculations. To the best of our knowledge, EPR signals in the geomagnetic field from the electron spins of transient pairs of radicals have not been observed in biochemical reactions. At the same time, the experience of spin chemistry, which agrees with the theory, indicates a faster spin relaxation at the conditions under discussion, about 1 ns. Perhaps, at the most, 10 ns. The magnetic effects fall in the same way, by a thousand or a hundred times, since they are roughly proportional to the spin relaxation time—unless the rate of chemical process is a “bottleneck” suppressing magnetic effects to an even greater extent. The correct value of the RPM effect is thus only 0.01–0.1% per 50 μT—a figure that follows from the fundamental relation γHτ∼1 [10] for ordinary values of MF 1–5 mT, at which noticeable magnetic effects occur [11]. Many experiments support this conclusion; e.g., it is in perfect agreement with a recent study [41].

Even if the spin relaxation time in radicals was about 1 μs, and even more so if noticeably less, the RPM in its usual form, as shown above, could not explain the specific sensitivity of some seasonally migratory species to geomagnetic variations in the MF at the level of tens of nT. There is also no explanation for often observed nonspecific effects of the geomagnetic storms on the state of organisms, e.g., [60]. This is a disappointing situation—we cannot explain these magnetic biological effects without reference to some obscure tricks of natural biological evolution.

What new findings can the above-described statistical mechanism bring to this state of affairs? First, the statistical amplification of initially weak magnetic RPM signals in the process of cellular translation makes it possible to raise the effect to the level of at least a few percent. These magnitudes are observable and verifiable. The results in Figure 1 indicate such effect sizes.

The statistical mechanism indicates a definite region in the biochemical machinery where the primary magnetic biological effects occur. This region is the active site of the tRNA–ribosome complex, where enzymatic processes of the recognition of cognate amino acids and their attachment to the growing protein chain take place. Thus, the statistical mechanism can also be validated by biochemical methods—in addition to testing the qualitative features that follow from the mathematical relation (4). Finally, we note that the magnetic influence on such a general molecular machine as the ribosome, which synthesizes many different proteins, is consistent with the experimentally observed fact that non-specific magnetic biological effects are mostly random effects [42], not allowing simple averaging.

In general, the amplification of the probability of local errors is valid for any process, the result of which would be error-free only if there were no errors at each step in a long series. In addition to translation, these could be replication and transcription. As is known, cells have developed surprisingly perfect mechanisms for repairing replication and transcription errors [43]. However, errors still occur at individual steps. Their low probability can accumulate and lead to cellular stress. Although relatively strong MFs of the order of several mT or more change the activity of, e.g., DNA polymerase [41], it is not yet known whether an MF of the geomagnetic level can influence the probability of *errors* in that enzyme.

## 5. Conclusions

The synthesis of protein molecules from a hundred or more units has been shown to occur with the accumulation of the probability of an error due to the stepwise cyclic process of cellular translation. This statistical amplification of local incorporation errors enhances the chance of translation failure and leads to a significant fraction of non-functional protein molecules. The amount of amplification is approximately proportional to the length of the synthesized biopolymer. The statistical accumulation of the probability of local errors in cellular translation is an autonomous phenomenon that helps to explain the biological effects of weak MFs.

The enzymatic process of recognition of amino acids and their addition to the synthesized molecule in cellular translation has been proposed to include the formation of intermediate pair of radicals with spin-correlated electrons. This makes sense because many enzymatic reactions that involve an electron trasfer are magnetically sensitive.

We present a mathematical model that combines the known mechanism of magnetic sensitivity of the intermediate pair of radicals, the RPM, and the statistical amplification mechanism described above. The model describes the changes in the probability of incorrectly synthesized molecules in response to a change in the weak MF of the geomagnetic field level. The model explains the biological effects of weak MFs without assuming an unlikely long spin relaxation time of 100 ns or more, often set by default to match the RPM models with the experiment.

The combined mechanism allows for experimental verification by testing qualitative features that match those of the RPM. The combined mechanism predicts the active center of a ribosome as the primary site, where magnetic effects occur, creating the possibility of verification by biochemical methods; predicts a random nature of the observed response to a weak MF; holds promise for explaining biological response to a hypomagnetic field; and agrees with the diversity of cell responses to a weak MF.

## Data Availability

Not applicable.

## Abbreviations

The following abbreviations are used in this manuscript:

DNA	deoxyribonucleic acid
EPR	electron paramagnetic resonance
mRNA	messenger ribonucleic acid
MF	magnetic field
RNA	ribonucleic acid
RPM	Radical Pair Mechanism
S-T	singlet-triplet

## Appendix A

### Appendix A.1. The RPM Chemical Kinetics

Various kinetic schemes for the RPM are studied in search of the nature of MF biological effects. Below we use an idealized scheme, which, on the one hand, is well-known in spin chemistry, e.g., [52]. On the other hand, we apply it to the biochemical process of the occurrence of errors in biopolymers in a general way—it works wherever such a process proceeds with the formation of a radical pair in a spin-correlated state,

(A1) $$B_{n}+A\;\overset{\alpha }{\underset{\beta }{⇄}}\;{\left(\mathrm{ABE}\right)}^{S}\;\overset{\omega }{\underset{\omega }{⇄}}\;{\left(\mathrm{ABE}\right)}^{T}\;\overset{k}{\to }\;B_{n+1}$$

where B${}_{n}$ is a biopolymer with *n* units, A is a unit link attached to the biopolymer, *not* being correct according to the blueprint specified in the case of cellular translation by mRNA. ABE is a complex of the enzyme E with the terminal site of the biopolymer B and the attached link A. We assume a pair of radicals with spin-correlated electrons occur in this complex, the state of which affects the probability of recognition of link A by the corresponding instruction. If the complex refuses to recognize link A as correct, A returns to the cytoplasm. If A is erroneously and with a low probability is recognized as correct, then A joins B${}_{n}$, and the polymer elongates incorrectly. Even if no more errors occur, the entirely synthesized and folded globule will no longer be fully functional.

Rate constants appear in the kinetic scheme: α is the rate of formation of the ABE complex and β is that of returning to the initial state, ω is the rate of S-T conversion, and *k* is that of occurring of the incorporation errors, or of attaching a non-cognate amino acid. The triplet states are depicted for convenience by one symbol. We assume the formation of the product only from the triplet state since this gives just a slightly larger effect size of the same order of magnitude while simplifying mathematics.

Denoting “substance concentrations” B${}_{n}$ and A, (ABE)${}^{S}$, (ABE)${}^{T}$, and B${}_{n+1}$ by *R* (right), *x*, *y*, and *F* (fault), respectively, we write the equations of chemical kinetics in the form

$$\begin{array}{ccc} \dot{x} & = & (\alpha R-\beta x)+\omega (y-x),\\ \dot{y} & = & -\omega (y-x)-ky \end{array}$$

The relative rate, *u*, of the incorporation errors, or the probability of a local failure per unit time, is $u\equiv \dot{F}/R=ky/R$. The stationary solution of the above system of equations gives the dependence of *u* on the model parameters,

(A2) $$u\left(\omega \right)=\frac{\alpha \omega k}{\beta \omega +\beta k+\omega k}$$

It is natural to require that the choice of parameters be consistent and lead to the values observed in the experiment. Then the number of parameters can be made smaller because α should be bound to other parameters.

Experimental data on folding errors are scarce [61]. Folding errors are more likely to be due to incorporation errors in the translation of the genetic code than to errors in actual folding and maturation of “correct” amino acid sequences [46]. Incorporation errors occur with a probability of the order of $q=10^{-4}$–$10^{-3}$ per peptide bond [45]. The synthesis rate, or that of elongation, of a protein chain is about 10–30 peptide bonds per second [62,63]. It means elongation by one peptide bond occurs in Δt∼ 1/20 s. Accordingly, the error rate $u_{0}\equiv q/\Delta t$ corresponding to the experimental data is $7\times 10^{-3}$ s${}^{-1}$ in order of magnitude. Although the error probability *q* may depend on the translated codon [44,64], the rough average of *q* is used below for estimates. Biochemical processes can regulate also the elongation time per bond, but it falls out of the final formulas.

The agreement with experiment is achieved by equating the experimental $u_{0}$ and theoretical (A2) rates, $u_{0}=u\left(\omega \right)$. The experimental value has been obtained in the geomagnetic field $H_{g}$, which means $u_{0}=u\left(\omega_{g}\right)$. Then from (A2) we get $\alpha =u_{0}\left[1+\beta \left(1/k+1/\omega_{g}\right)\right]$. Substituting this α value back into (A2) one can find

(A3) $$u\left(\omega \right)=u_{0}\frac{1+j/\omega_{g}}{1+j/\omega },\;\;\frac{1}{j}=\frac{1}{k}+\frac{1}{\beta }$$

Parameters *k* and β affect *u* through their combination *j* only. Therefore, further in calculations we use this harmonic mean *j* instead of *k* and β separately.

### Appendix A.2. The Rate of a S-T Conversion in the MF

There are several ways in which an external MF can cause a magnetochemical effect. We consider one suitable for weak MF, less than 1 mT—the so-called low field effect, e.g., [52]. In a simplified version of this mechanism, there are a pair of electrons and a magnetic nucleus near one of them. The hyperfine interaction causes a change in the spin state of this electron, and a S-T conversion of the spin state of the pair occurs. External MF slightly alters the conversion speed. This model does not allow for the interaction of electrons, nor, usually, several magnetic nuclei acting on electrons, nor the movement of radicals. Therefore, it gives exaggerated magnitudes of magnetic effects [65]. However, the model makes it possible to estimate the maximum possible MF effect magnitudes.

In [52] Equation (12), it was shown that under some idealizing assumptions, in particular for isotropic hyperfine interaction in the immobile radical pair and small chemical rate constant *k*, the time-averaged maximum relative loss in the singlet product, or roughly growth of the triplet product, of S-T conversion in the RPM can be written as

(A4) $$w=\frac{5}{8}-\frac{1}{4+{\left(\gamma H\tau \right)}^{2}}$$

where the characteristic time of formation of the S-T conversion products is replaced by thermal relaxation time τ, which is the shortest of the two times and, therefore, is a “bottleneck” of the conversion process. We use this simplified expression for *w* to demonstrate the quadratic functional relationship between the MF intensity and the incorporation error probability, which is then enhanced during cellular translation. The quadratic effect in the region of small MFs up to 1 mT was observed experimentally in, e.g., [66,67,68,69,70,71,72] and evaluated theoretically in, e.g., [52,73,74,75].

The change in the amount *w*, or probability, of the triplet product, with which magnetic effects based on the RPM are often associated, can be relatively large for large values of the argument. However, this is not the case in the MF of the geomagnetic field level at the expected spin relaxation time τ∼1 ns, because $\gamma H\tau ∼10^{-3}$–$10^{-2}$. Then the usual RPM-based explanation of the MF effect fails. This explanation of biological magnetic effects survives when one considers the amplification of weak magnetic signals in cellular translation. Note again that Equation (A4) is a rather rough idealization for small τ, and its use is only illustrative. Due to the smallness of ${\left(\gamma H\tau \right)}^{2}$, the triplet product probability can be written as a proportion

(A5) $$w\left(h,\tau \right)\propto a+z\left(\tau \right)h^{2}$$

where *a* is a constant, $h\equiv H/H_{g}$ and $z\left(\tau \right)\equiv {\left(\gamma H_{g}\tau \right)}^{2}$. This form of the product probability is more general than (A4) and will be used later. The average rate of S-T conversion over time τ then is ω=w/τ.
